# Relation of Prostatic and Urinary Bladder Ultrasound Parameters in Patients With Benign Prostatic Hyperplasia

**DOI:** 10.1002/hsr2.72195

**Published:** 2026-03-30

**Authors:** Bhishma Prasad Pokharel, Narayan Bikram Thapa, Jeni Singh, Yashoda Dangi, Dip Bahadur Singh

**Affiliations:** ^1^ Kathmandu Medical College Teaching Hospital Kathmandu Kathmandu University; ^2^ Asian College for Advance Studies Purbanchal University Nepal; ^3^ Valley College of Technical Sciences Purbanchal University Nepal; ^4^ Department of Health Informatics, School of Engineering Kathmandu University Nepal

**Keywords:** benign prostatic hyperplasia, bladder wall thickness, intravesical prostatic protrusion, lower urinary tract symptoms, post‐void residual urine, prostate volume, ultrasonography

## Abstract

**Background:**

Benign prostatic hyperplasia (BPH) is a prevalent condition among aging males, leading to progressive lower urinary tract symptoms (LUTS) due to prostate enlargement and bladder dysfunction. This study aimed to assess the correlation between prostate volume (PV) and key ultrasonographic parameters, including intravesical prostatic protrusion (IPP), bladder wall thickness (BWT), and post‐void residual urine (PVRU), to evaluate their clinical significance in BPH management.

**Methods:**

A cross‐sectional study was conducted on 105 male patients diagnosed with BPH. Prostate volume, IPP, BWT, and PVRU were measured using transabdominal ultrasonography. Pearson's correlation analysis and chi‐square tests were used to assess the associations between these parameters. Statistical significance was set at *p* < 0.05.

**Results:**

Strong positive correlation were observed between PV and IPP (*r* = 0.789, *p* < 0.001), PV and BWT (r = 0.65, *p* < 0.001), and PV and PVRU (*r* = 0.805, *p* < 0.001). The highest correlation was found between IPP and PVRU (*r* = 0.851, *p* < 0.001), suggesting that increased IPP is a strong predictor of urinary retention. Patients with larger prostates ( > 60 cc) exhibited significantly higher grades of IPP, BWT, and PVRU (*p* < 0.001), indicating a progressive decline in bladder function as prostate volume increases.

**Conclusion:**

This study confirms that IPP, BWT, and PVRU are strongly associated with prostate volume, with IPP emerging as a key predictor of urinary retention severity in patients with BPH. These findings underscore the importance of integrating ultrasonographic assessments, particularly IPP and BWT measurements, into routine clinical evaluations to improve the early detection and management of BPH. Future multicenter, longitudinal studies are recommended to validate these findings and optimize clinical decision‐making.

## 1 Introduction

Benign Prostatic Hyperplasia (BPH) is a prevalent non‐malignant enlargement of the prostate gland that leads to lower urinary tract symptoms (LUTS) in aging men. Diagnosis is typically based on a combination of clinical symptoms and radiological findings, with a prostate volume ≥ 25 cc considered a diagnostic threshold. Prostate cancer is commonly ruled out by digital rectal examination (DRE) and serum prostate‐specific antigen (PSA) testing. While current guidelines rely heavily on symptom scoring systems and urodynamic studies, ultrasonographic parameters such as intravesical prostatic protrusion (IPP), bladder wall thickness (BWT), and post‐void residual urine (PVRU) have emerged as promising noninvasive predictors of bladder outlet obstruction (BOO). IPP has been shown to be significantly associated with disease progression in BPH patients [1]. Similarly, strong correlations between IPP, BWT, and symptom severity have been reported by Ham and Sigdel [2, 3].

Although current international guidelines for benign prostatic hyperplasia (BPH) emphasize the use of symptom scores such as the International Prostate Symptom Score (IPSS) and urodynamic studies for assessing bladder outlet obstruction (BOO), they do not yet fully incorporate ultrasonographic markers such as intravesical prostatic protrusion (IPP), bladder wall thickness (BWT), and post‐void residual urine (PVRU) into standard diagnostic protocols. Emerging evidence suggests that these parameters offer reliable, non‐invasive insight into BOO severity and correlate more strongly with patient outcomes than prostate volume alone [2, 4]. Despite their diagnostic potential, these sonographic markers remain underutilized, warranting further validation and clinical integration.

### 1.1 Global Scenario

Ultrasound parameters such as IPP, BWT, and PVRU have emerged as non‐invasive predictors of BOO in BPH [5]. PP, in particular, correlates with disease progression and trial without catheter success [6]. Metabolic syndrome components are also linked to prostate enlargement and LUTS [7]. The epidemiology and etiology of BPH highlight modifiable risk factors, including obesity, metabolic syndrome, inflammation, and diet [8]. Despite this, these sonographic markers remain underutilized in clinical guidelines.

### 1.2 South Asian Scenario

In South Asia, age is a key factor in BPH incidence, with most cases occurring in the 61–70 age group [9]. Prostatic inflammation in patients with BPH is linked to lower Qmax and higher preoperative prostate scores, highlighting its role in disease progression [10]. A previous study found a significant correlation between prostate volume and symptom severity (IPSS) in patients with BPH [11]. The International Prostate Symptom Score (IPSS) effectively categorizes BPH severity and guides treatment [12]. However, comprehensive ultrasound‐based assessments are lacking in regional studies.

### 1.3 Objectives

This study aims to assess the relationship between prostatic and urinary bladder ultrasound parameters in patients with benign prostatic hyperplasia (BPH). It focuses on measuring prostatic parameters via transabdominal ultrasound, evaluating bladder wall thickness in a filled urinary bladder, and analyzing their correlation. Establishing these associations that enhance the diagnostic and prognostic value of ultrasound in BPH management.

## 2 Methodology

### 2.1 Ethical Considerations

This study was approved by the Institutional Review Committee Ref. No: 0308202104. Informed consent was obtained from all the patients, and data confidentiality was ensured.

### 2.2 Study Design

This study used a cross‐sectional design to assess the correlation between prostatic and bladder ultrasound parameters in BPH patients.

### 2.3 Study Population and Setting

This study was conducted at a tertiary care hospital in Nepal and included male patients aged ≥ 40 years who underwent transabdominal ultrasonography between September 2021 and October 2022. The hospital's diverse patient population provides a representative clinical sample. All participants were diagnosed with benign prostatic hyperplasia (BPH) based on a combination of clinical presentation and radiological assessment. Specifically, BPH was defined by the presence of lower urinary tract symptoms (LUTS), such as frequency, urgency, nocturia, and weak urinary stream, along with a prostate volume ≥ 25 cc as measured by transabdominal ultrasound, consistent with established diagnostic thresholds [4, 13]. Prostate cancer was ruled out through digital rectal examination (DRE) and serum prostate‐specific antigen (PSA) testing in accordance with standard urological protocols. This comprehensive approach ensured the inclusion of symptomatic patients with BPH with no evidence of malignancy.

### 2.4 Sampling Method and Sample Size Calculation

A non‐random, convenience sampling method was used to select eligible participants. The sample size was determined based on a previous retrospective study by Baidya et al. [14], which reported a prevalence of BPH at 45.45% (5 out of 11 cases). The minimum required sample size was calculated using the *Z*²*pq*/*d*² formula for proportion‐based sample estimation: *n* = *Z*²*pq*/*d*².

where *Z* = 1.96 (95% confidence level), *p* = 0.4545 (prevalence), *q* = 1 − *p* = 0.5455, and *d* = 0.1 (10% margin of error). The calculation yielded *n* = 95.2, which was rounded up to 105 participants after adjusting for a 10% non‐response rate.

### 2.5 Eligibility Criteria

**Inclusion criteria:** Adult males ( ≥ 40 years) with prostate volume ≥ 25 cc who underwent transabdominal sonography at a tertiary care hospital, **Exclusion criteria:** included a history of lower urinary tract or pelvic surgery, pelvic trauma, diabetic cystopathy, neurological bladder dysfunction, bladder stones, cancer, inability to fill the bladder to ≥ 200 mL, or lack of consent.

### 2.6 Study Variables

This study analyzed prostate volume, IPP, BWT, and PVRU as dependent variables, with age as the independent variable.

### 2.7 Data Collection Methods and Measurement Tools

Data were prospectively collected using transabdominal ultrasonography with a 2–8 MHz convex probe on a Canon Toshiba Aplio 400 machine. The prostate volume (PV) was calculated using the ellipsoid formula (PV = π/6 × width × height × length). Intravesical prostatic protrusion (IPP), bladder wall thickness (BWT), and post‐void residual urine (PVRU) were measured in the midsagittal view. Variables were categorized as PV (25–40 cc, 40–60 cc, > 60 cc), IPP (Grade I: < 5 mm, Grade II: 5–10 mm, Grade III: > 10 mm), BWT (Grade I: < 5 mm, Grade II: ≥ 5 mm), and PVRU ( < 50 mL, 50–100 mL, > 100 mL).

### 2.8 Bias and Missing Data Management

To reduce bias, standardized eligibility criteria were applied, ultrasound was performed by the same radiologist, and the equipment was calibrated using a validated protocol. Missing data were handled using mean imputation for continuous variables and listwise deletion for categorical data.

### 2.9 Data Management and Statistical Analysis

The data were analyzed using Python 3.12.7. Continuous variables were summarized as mean ± standard deviation (SD), and categorical variables as frequencies and percentages. Associations were assessed using chi‐square tests, group comparisons with one‐way ANOVA, and correlations with the Pearson's *r* test. Statistical significance was set at *p* < 0.05 (two‐sided). Libraries used included pandas (v2.1.1), numpy (v1.26.0), scipy. stats (v1.11.3), matplotlib (v3.8.0), seaborn (v0.13.0), and statsmodels (v0.14.0).

## 3 Results

### 3.1 Study Population Characteristics

A total of 105 male BPH patients (aged 40–90, mean age: 63.55 ± 13.90) were included, with no missing data. Statistical analyses were performed using descriptive and inferential methods to ensure accurate and unbiased reporting.

### 3.2 Participant Flow Diagram

**Figure 1:**
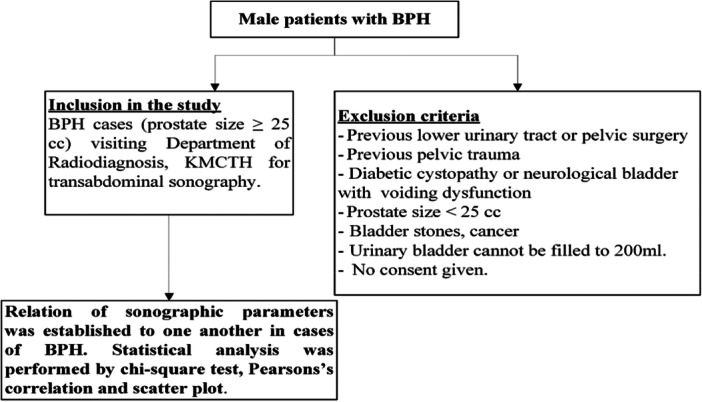
Participants selection flow diagram.

The participant selection process is illustrated in Figure 1. Inclusion criteria: BPH patients with a prostate size ≥ 25 cc who underwent transabdominal ultrasonography at a tertiary care hospital. Exclusion criteria were a history of lower urinary tract surgery, pelvic trauma, neurological bladder dysfunction, bladder stones, cancer, inability to fill the bladder to ≥ 200 mL, and lack of consent. The final sample size was determined after applying these criteria.

### 3.3 Descriptive Statistics of Study Participants

Table 1 presents the descriptive statistics of 105 male patients, including age, prostate volume (PV), bladder wall thickness (BWT), post‐void residual urine (PVRU), and intravesical prostatic protrusion (IPP).

**Table 1:** Descriptive statistics of study participants (*n* = 105).

Variable	Mean (*M*)	Standard deviation (SD)	Range (min–max)	Unit of measurement
Age	63.55	13.90	40–90	Years
Prostate Volume (PV)	42.75	16.82	25–106	cc
Bladder Wall Thickness (BWT)	5.33	2.00	2–12	mm
Post‐Void Residual Urine (PVRU)	59.30	46.01	0–180	mL
Intravesical Prostatic Protrusion (IPP)	5.56	5.60	0–23	mm

The mean age of the participants was 63.55 years (SD = 13.89, range: 40–90 years). Prostate volume ranged from 25 cc to 106 cc, with a mean of 42.75 cc (SD = 16.82). Other key urological parameters included BWT, PVRU, and IPP.

### 3.4 Association Between Age and Prostate Volume

Table 2 shows the relationship between age and prostate volume (PV). The participants were categorized into five age groups ( < 50, 50–60, 60–70, 70–80, and > 80 years), and their prostate volume was classified into three categories (25–40 cc, 40–60 cc, and > 60 cc). Table 2 presents these findings.

**Table 2:** Association between age group and prostate volume.

Age group	Prostate volume (cc)				*Χ*²	*p* value*
	25–40	40–60	> 60	Total		
< 50	22 (100.0%)	0 (0.0%)	0 (0.0%)	22	46.924	< 0.001*
50–60	16 (72.7%)	6 (27.3%)	0 (0.0%)	22		
60–70	8 (36.4%)	12 (54.5%)	2 (9.1%)	22		
70–80	11 (50.0%)	4 (18.2%)	7 (31.8%)	22		
> 80	3 (17.6%)	7 (41.2%)	7 (41.2%)	17		

A statistically significant association (χ² = 46.924, *p* < 0.001) was observed between age and prostate volume, indicating that prostate enlargement progresses with age. Older patients (> 70 years) had a significantly higher proportion of prostate volumes exceeding 60 cc, whereas younger individuals (< 60 years) predominantly had prostate volumes in the range of 25–40 cc. These findings emphasize the age‐related progression of benign prostatic hyperplasia (BPH), reinforcing the necessity of early screening and monitoring to facilitate timely intervention and reduce the risk of BPH‐related complications. See plot in Supporting figure 1.

### 3.5 Correlation Between Prostate Volume, IPP, BWT, and PVRU

Table 3 presents the correlation coefficients from Pearson's correlation analysis, which was conducted to assess the strength of the relationships among age, prostate volume (PV), IPP, BWT, and PVRU. Statistically significant associations (*p* < 0.001) between prostate volume and key urinary parameters.

**Table 3:** Correlation between urological parameters.

Variables	Correlation coefficient (*r*)	95% CI	*p*‐value
Age and PV	0.526	(0.37–0.65)	< 0.001**
PV and IPP	0.789	(0.7–0.85)	< 0.001**
PV and BWT	0.65	(0.52–0.75)	< 0.001**
PV and PVRU	0.805	(0.72–0.86)	< 0.001**
IPP and BWT	0.739	(0.64–0.82)	< 0.001**
IPP and PVRU	0.851	(0.79–0.9)	< 0.001**

These findings indicate that as prostate size increases, the severity of bladder dysfunction also worsens. The strongest correlation was observed between IPP and PVRU (*r* = 0.85), indicating that severe intravesical prostatic protrusion is highly predictive of urinary retention. See plot in Supporting figure 2.

### 3.6 Association Between Prostate Volume and Key Urological Parameters

Table 4 evaluates the impact of prostate volume (PV) on urinary function, we analyzed its correlation with intravesical prostatic protrusion (IPP), bladder wall thickness (BWT), and post‐void residual urine (PVRU). Table 3 presents these associations.

**Table 4:** Association between prostate volume with IPP, BWT and PVRU.

Variables		Prostate volume(cc)			Total	*Χ²*	p value*
		25–40	40–60	> 60			
IPP	Grade I	48 (92.3%)	4 (7.7%)	0 (0.0%)	52	92.522	< 0.001*
	Grade II	12 (36.4%)	19 (57.6%)	2 (6.1%)	33		
	Grade III	0 (0.0%)	6 (30.0%)	14 (70.0%)	20		
BWT	Grade I	45 (91.8%)	4 (8.2%)	0 (0.0%)	49	45.944	< 0.001*
	Grade II	15 (26.8%)	25 (44.6%)	16 (28.6%)	56		
PVRU	< 50	45 (91.8%)	4 (8.2%)	0 (0.0%)	49	77.992	< 0.001*
	50–100	15 (41.7%)	18 (50.0%)	3 (8.3%)	36		
	> 100	0 (0.0%)	7 (35.0%)	13 (65.0%)	20		

A statistically significant correlation (*p* < 0.001) was observed between prostate volume and IPP, BWT, and PVRU. Patients with Grade III IPP, Grade II BWT, and PVRU > 100 mL had larger prostates (> 60 cc), while those with Grade I IPP, Grade I BWT, and PVRU < 50 mL had smaller prostates (25–40 cc). These findings confirm that increasing prostate volume is associated with greater urinary obstruction and retention, emphasizing the clinical importance of these parameters in BPH management. See plot in Supporting Figures 3, 4, 5.

### 3.7 Scatter Plot Showing Relationship Between IPP and PVRU

**Figure 2:**
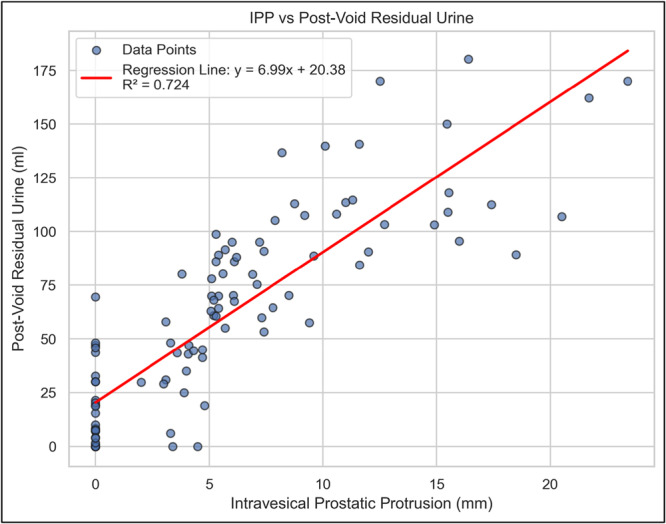
Scatter plot showing the relationship between IPP and PVRU.

Figure 2 presents the correlation between Intravesical Prostatic Protrusion (IPP) and Post‐Void Residual Urine (PVRU) in patients diagnosed with Benign Prostatic Hyperplasia (BPH). The scatter plot reveals a strong positive correlation, indicating that an increase in IPP is associated with a progressive rise in PVRU levels.

The regression equation (y = 20.38 + 6.99x) suggests that for each unit increase in IPP (mm), the PVRU (mL) increases by an average of 6.99 mL. The coefficient of determination (*R*² = 0.724) implies that 72.4% of the variation in PVRU can be explained by changes in IPP, underscoring the potential of IPP as a predictor of urinary retention severity in BPH patients. Even at 0 IPP, there is significant PVRU, as PVRU also has a positive correlation with age, prostate size, and bladder wall thickness. These findings reinforce the clinical importance of IPP measurement in assessing bladder dysfunction and guiding BPH management strategies.

### 3.8 Relation Between IPP Grade With BWT and PVRU

Table 5 depicts the relationship between intravesical prostatic protrusion (IPP) grades and bladder wall thickness (BWT) and post‐void residual urine (PVRU). The data reveals that higher IPP grades are significantly associated with increased PVRU and BWT, suggesting a progressive worsening of urinary retention and bladder changes with increasing prostate protrusion.

**Table 5:** Association between IPP grade, BWT and PVRU.

Variables		IPP grade			Total	*Χ*²	*p* value*
		Grade I n (%)	Grade II *n* (%)	Grade III *n* (%)			
PVRU (mL)	< 50	49 (100.0%)	0 (0.0%)	0 (0.0%)	49	140.857	< 0.001*
	50–100	3 (8.3%)	29 (80.6%)	4 (11.1%)	36		
	> 100	0 (0.0%)	4 (20.0%)	16 (80.0%)	20		
BWT	Grade I	49 (100.0%)	0 (0.0%)	0 (0.0%)	49	93.642	< 0.001*
	Grade II	3 (5.4%)	33 (58.9%)	20 (35.7%)	56		

The statistically significant correlation (*p* < 0.001) between IPP grades and both PVRU and BWT is shown in Supporting figures 6 and 7. Patients with Grade III IPP predominantly had PVRU > 100 mL and Grade II BWT, whereas those with Grade I IPP had PVRU < 50 mL and Grade I BWT. This suggests that greater intravesical protrusion is linked to higher post‐void residual urine and increased bladder wall thickness, reinforcing its importance in assessing BPH severity and urinary dysfunction.

## 4 Discussion

This study investigated the correlation between prostate volume (PV) and three ultrasonographic parameters, intravesical prostatic protrusion (IPP), bladder wall thickness (BWT), and post‐void residual urine (PVRU), in patients with symptomatic benign prostatic hyperplasia (BPH). These findings support a strong and statistically significant association between PV and these ultrasound‐based variables, reinforcing their relevance in BPH evaluation. The mean patient age was 63.55 years, aligning with findings that BPH mainly affects men over 60 years of age [4, 15]. The mean prostate volume was 42.74 cc, consistent with studies showing average volumes of 40–50 cc in similar populations [2].

The strong correlation between PV and IPP (*r* = 0.789) reflects previous evidence that IPP is a more reliable predictor of bladder outlet obstruction (BOO) than PV alone [16, 17]. Lim et al. also associated elevated IPP with the clinical progression of BPH [4]. The correlation between PV and BWT (*r* = 0.65) suggests detrusor hypertrophy due to increased resistance, which is in agreement with studies showing bladder wall thickening as an adaptive response [9].

PV and PVRU were significantly correlated (*r* = 0.80), supporting earlier reports of impaired bladder emptying in patients with larger prostates [6]. Notably, IPP and PVRU demonstrated the strongest correlation (*r* = 0.851), reinforcing IPP's roleas a primary anatomical contributor to urinary retention [17]. Additional correlations among IPP, BWT (*r* = 0.739), and PVRU (*r* = 0.851) confirm the interdependence between structural and functional changes in BPH [18].

Despite robust evidence, IPP, BWT, and PVRU remain underutilized in diagnostic guidelines. Their inclusion could improve BPH assessment, especially in settings that lack invasive urodynamic testing. The limitations of this study include its single‐center design and cross‐sectional nature. Larger prospective studies are recommended to confirm these findings and establish diagnostic thresholds.

In conclusion, IPP, BWT, and PVRU were significantly correlated with prostate volume in symptomatic BPH. IPP showed the strongest association with urinary retention, underscoring its diagnostic value.

## 5 Conclusion

This study highlights the strong association between prostate volume (PV) and urinary dysfunction parameters in hyperplasia (BPH) patients. Intravesical prostatic protrusion (IPP) and post‐void residual urine (PVRU) showed the strongest correlation (*r* = 0.85), with IPP emerging as a key predictor of urinary retention. As the prostate size increased, higher IPP grades, thicker bladder walls, and elevated PVRU were observed, indicating worsening urinary dysfunction. These findings emphasize the clinical relevance of ultrasonographic parameters in assessing BPH progression and identifying patients at risk of severe bladder dysfunction. The study recommends integrating IPP and bladder wall thickness assessments into routine evaluations, prioritizing early screening, and implementing structured interventions to prevent complications, such as acute urinary retention and detrusor dysfunction.

## 6 Future Research and Limitations

Future research should focus on longitudinal studies to assess disease progression, and intervention‐based approaches with urodynamic assessments for validation. The single‐center design of this study limits generalizability, and a larger multicenter cohort is needed for broader applicability. Additionally, the cross‐sectional design restricts causal inference, and external factors such as comorbidities and medication effects have not been thoroughly examined.

## Author Contributions

**Bhishma Prasad Pokharel:** conceptualization, methodology, investigation, formal analysis, data curation, validation, visualization, writing – original draft. **Narayan Bikram Thapa:** data curation, validation, visualization, writing – review and editing. **Jeni Singh:** validation, visualization, writing – review and editing. **Yashoda Dangi:** validation, visualization, writing – review and editing. **Dip Bahadur Singh:** conceptualization, formal analysis, validation, project administration, funding acquisition, writing – review and editing.

## Funding

The authors have nothing to report.

## Disclosure

All authors have read and approved the final version of the manuscript. Dr. Bhisma Prasad Pokharel, Prof. Dr. Narayan Bikram Thapa, Dip Bahadur Singh, Jeni Singh and Yashoda Dangi had full access to all of the data and takes complete responsibility for its integrity and accuracy.

## Conflicts of Interest

The authors declare that they have no known competing financial interests or personal relationships that could have appeared to influence the work reported in this paper.

## AI Use

The authors confirm that no generative AI tools were used in the writing, editing, or data analysis of this manuscript. All content was prepared, reviewed, and finalized solely by the authors, who take full responsibility for its accuracy and integrity.

## Transparency Statement

The lead author, Dip Bahadur Singh, affirms that this manuscript is an honest, accurate, and transparent account of the study being reported; that no important aspects of the study have been omitted; and that any discrepancies from the study as planned (and, if relevant, registered) have been explained.

## Supporting information

Figure_1_Age_vs_ProstateVolume.

Figure_2_IPP_vs_PVRU.

Figure_3_Correlation_Matrix.

Figure_4_IPP_vs_ProstateVolume.

Figure_5_BWT_vs_ProstateVolume.

Figure_6_PVRU_vs_ProstateVolume.

Figure_7_BWT_vs_IPP.

Supplementary Information

## Data Availability

All relevant data are within the manuscript. Additional information can be obtained upon request from the corresponding author.
