# Supplementary figures and images for: Relation of Prostatic and Urinary Bladder Ultrasound Parameters in Patients With Benign Prostatic Hyperplasia
Source: Health Sci Rep. 2026 Mar 30;9(4):e72195. doi: 10.1002/hsr2.72195 (PMC13087513; doi:10.1002/hsr2.72195)

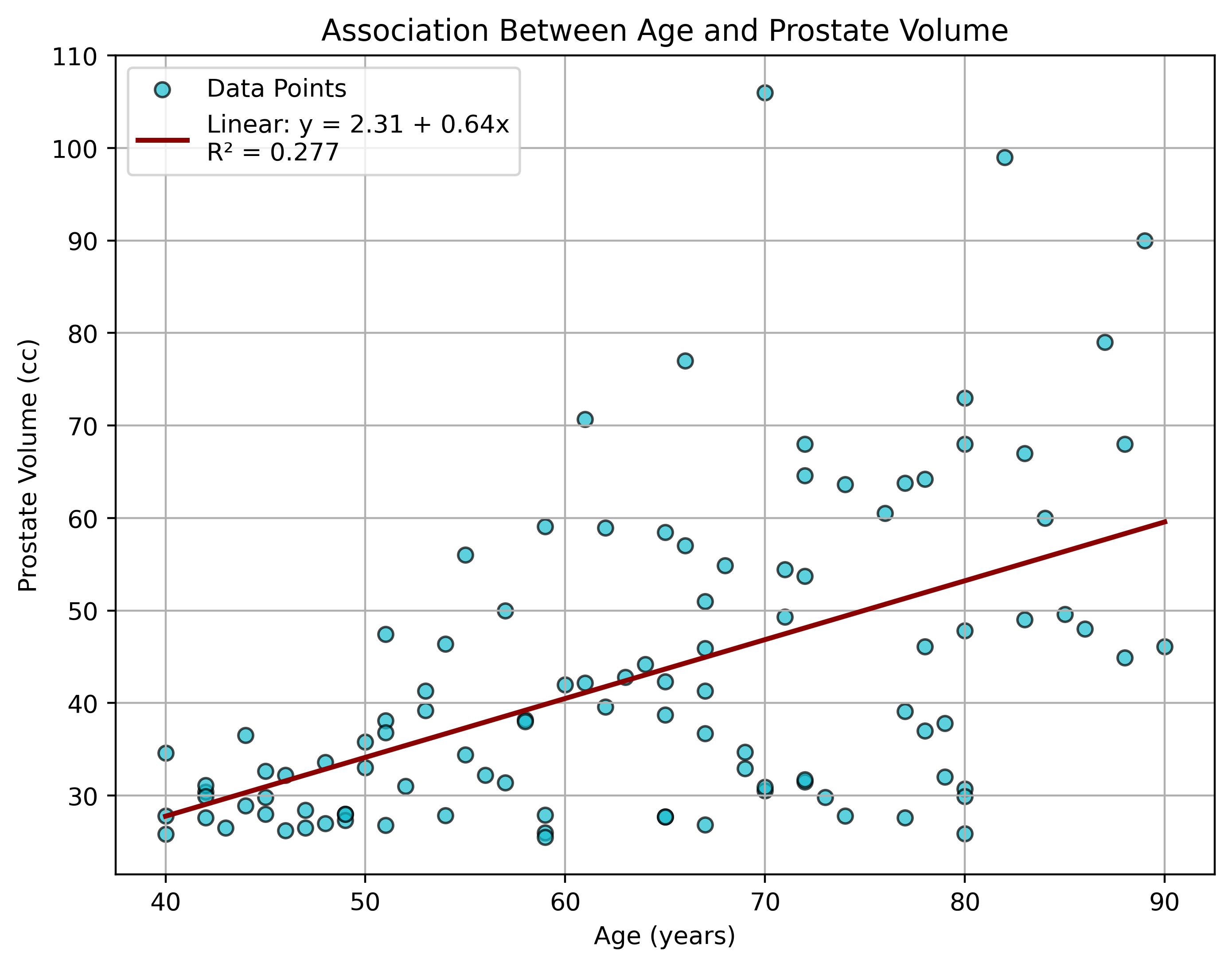

Supplement: Supplementary file 1 — Figure_1_Age_vs_ProstateVolume. [file HSR2-9-e72195-s008.tif]

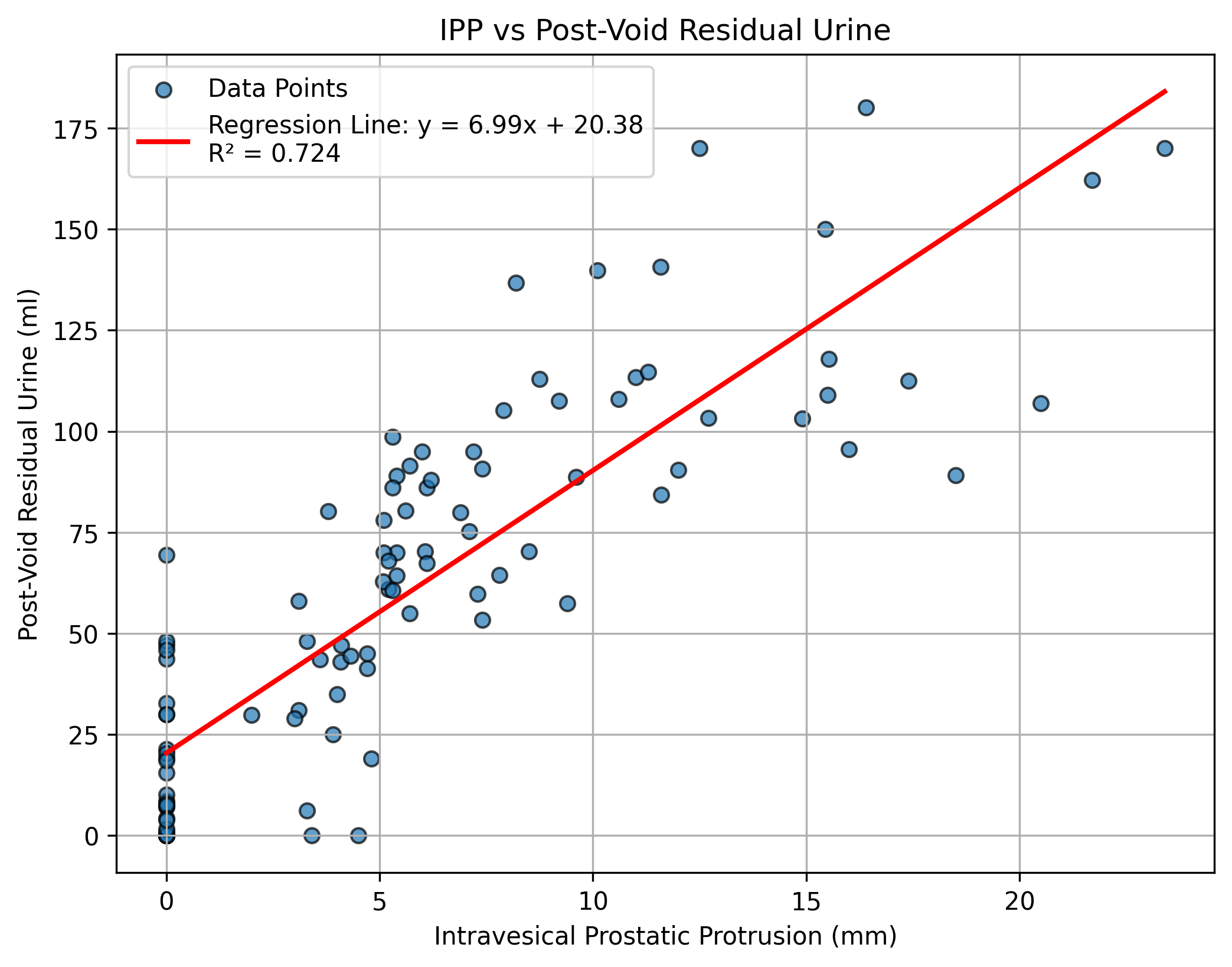

Supplement: Supplementary file 2 — Figure_2_IPP_vs_PVRU. [file HSR2-9-e72195-s005.tif]

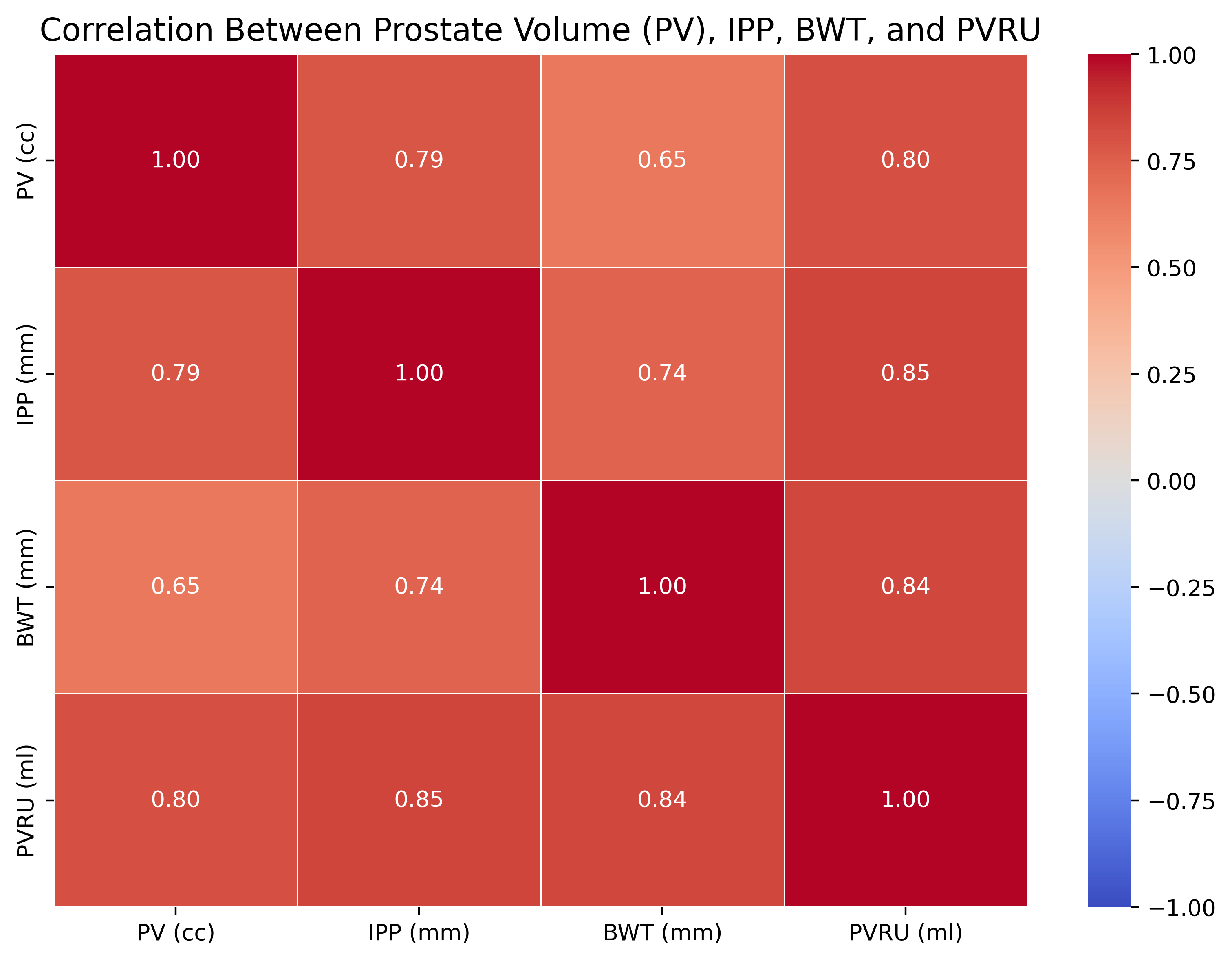

Supplement: Supplementary file 3 — Figure_3_Correlation_Matrix. [file HSR2-9-e72195-s001.tif]

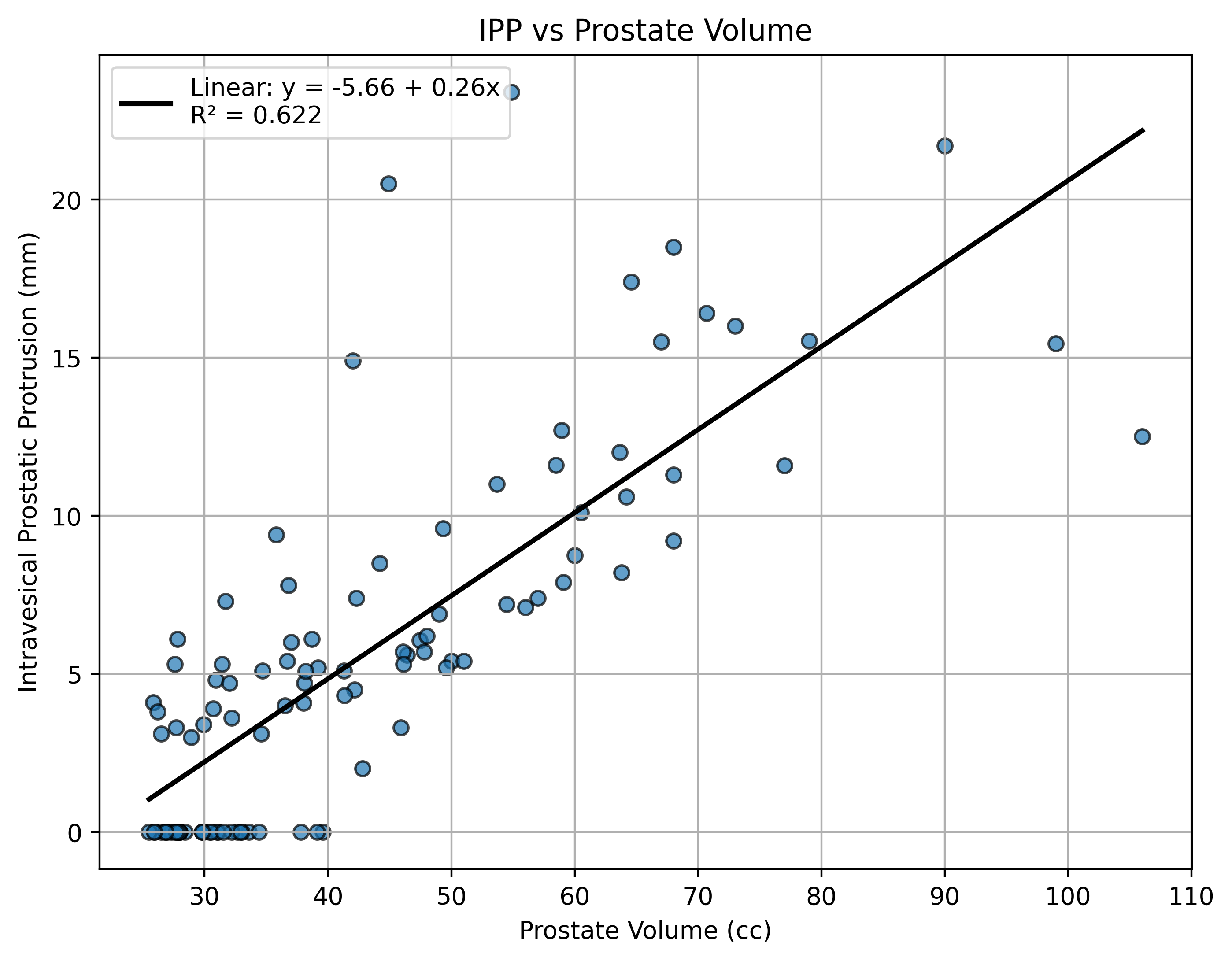

Supplement: Supplementary file 4 — Figure_4_IPP_vs_ProstateVolume. [file HSR2-9-e72195-s006.tif]

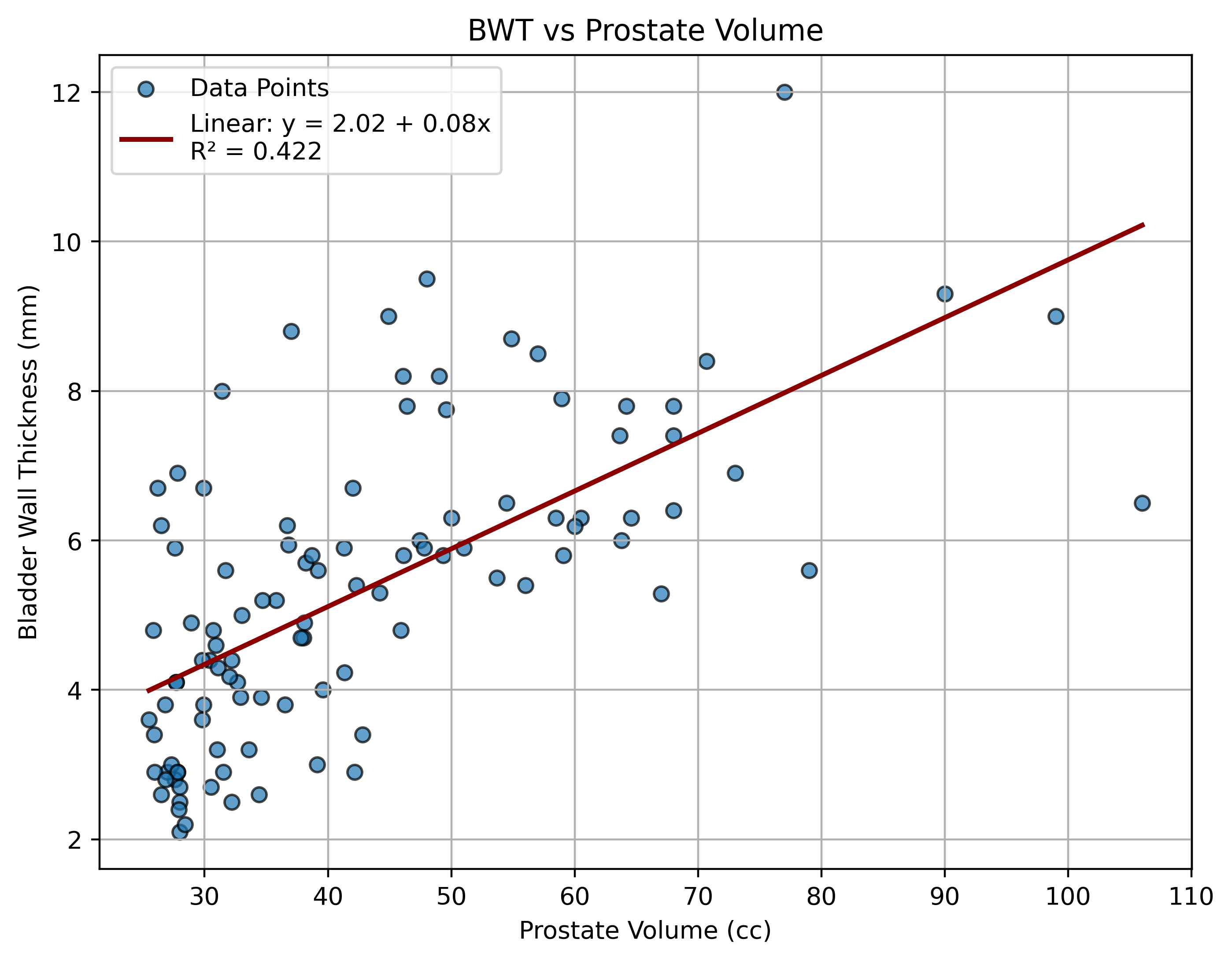

Supplement: Supplementary file 5 — Figure_5_BWT_vs_ProstateVolume. [file HSR2-9-e72195-s002.tif]

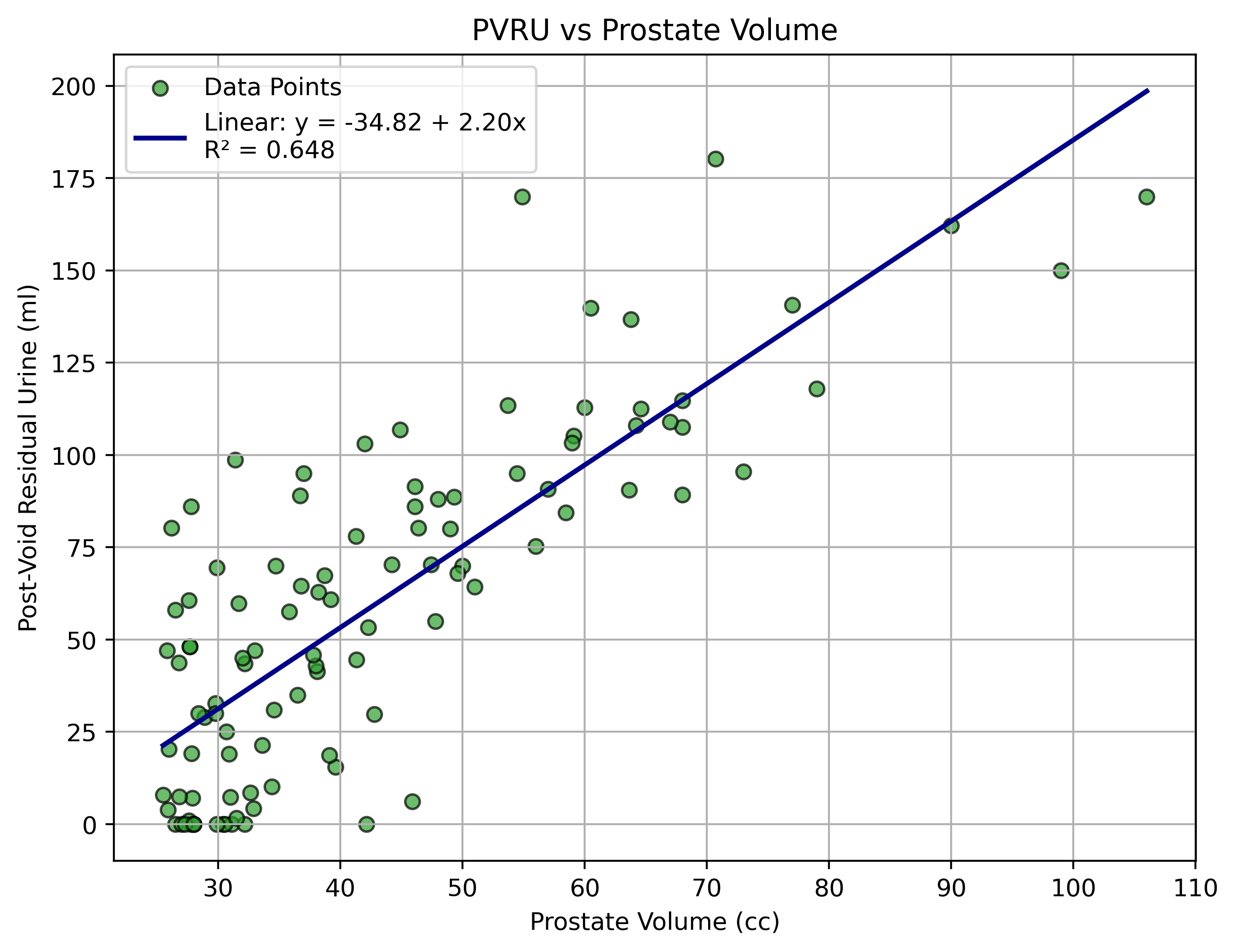

Supplement: Supplementary file 6 — Figure_6_PVRU_vs_ProstateVolume. [file HSR2-9-e72195-s004.tif]

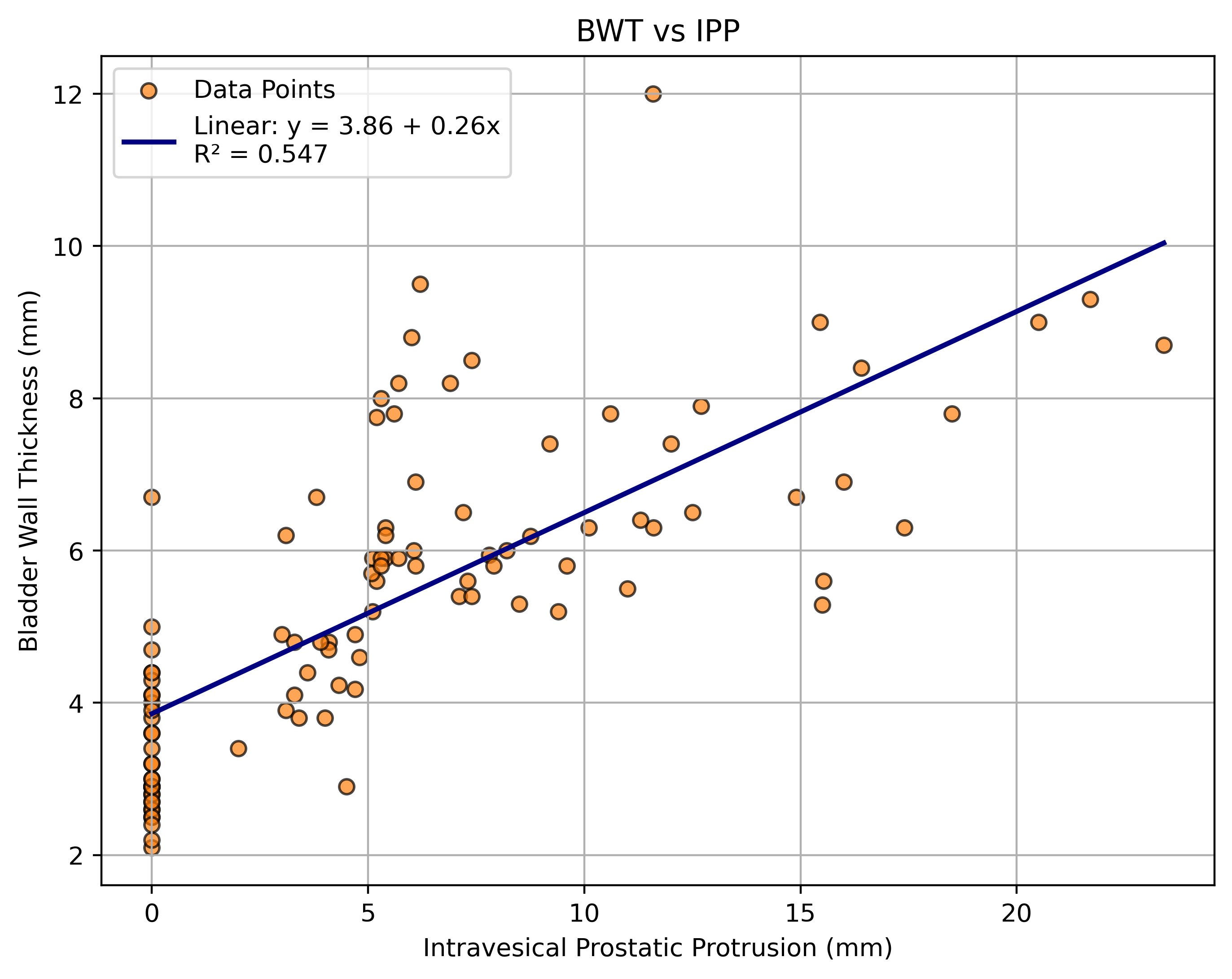

Supplement: Supplementary file 7 — Figure_7_BWT_vs_IPP. [file HSR2-9-e72195-s003.tif]
