# Supplementary material for: Relation of Prostatic and Urinary Bladder Ultrasound Parameters in Patients With Benign Prostatic Hyperplasia
Source: Health Sci Rep. 2026 Mar 30;9(4):e72195. doi: 10.1002/hsr2.72195 (PMC13087513; doi:10.1002/hsr2.72195)
Supplement: Supplementary file 8 — Supplementary Information [file HSR2-9-e72195-s007.docx]

**3.4. Association Between Age and Prostate Volume**
See Supplementary Figure 1.

**3.5. Correlation Between Prostate Volume, IPP, BWT, and PVRU**
See Supplementary Figure 3.

**3.6. Association Between Prostate Volume and Key Urological Parameters**
See Supplementary Figures 4, 5, and 6.

**3.8. Relationship Between IPP Grade, BWT, and PVRU**
See Supplementary Figure 7.
